# Infrequency of Phthisis in Marshy Districts

**Published:** 1844-03-23

**Authors:** 


INFREQUENCY OF PHTHISIS IN MARSHY DISTRICTS.

M. Ollivier (d'Angers), at the séance of the Academie de Médicine, Nov. 7, said that M. Nepple's researches in the infrequency of phthisis in marshy districts, were confirmed by what M. Brera said some years ago on the rarity of phthisis at Venice, a rarity which he attributed to the emanations of the lagunes. During a sojourn at Venice he endeavoured to verify this fact, and found that in the 1,200 or 1,400 patients admitted into the hospitals of that town in a year, there were only seven or eight cases of phthisis, the rest being for the most part cases of intermittent fever and rheumatism.—*Prov. Med. Jour. from Gaz. des Hop.*

